# Vibration assisted analgesia during intralesional corticosteroid therapy for alopecia

**DOI:** 10.1002/ski2.363

**Published:** 2024-03-18

**Authors:** Matthew D. Wynne, Thomas Harries, Charlotte Hennegan, Nuala O’Donoghue, Donna M. Cummins, Matthew Harries

**Affiliations:** ^1^ Salford Royal Hospital Northern Care Alliance NHS Foundation Trust Manchester Academic Health Science Centre Manchester UK; ^2^ Newcastle University School of Medicine Newcastle upon Tyne UK; ^3^ Faculty of Biology, Medicine and Health Centre for Dermatology Research University of Manchester Manchester UK

## Abstract

Intralesional corticosteroid therapy (ICT) is a recommended management strategy for various inflammatory hair loss disorders. Pain from ICT can limit the use of this treatment, particularly in younger people and those with needle phobia. We present data demonstrating that vibration assisted analgesia, is a safe, effective and easy to use technique, which minimises pain from ICT, allowing its use in a wider cohort of patients.

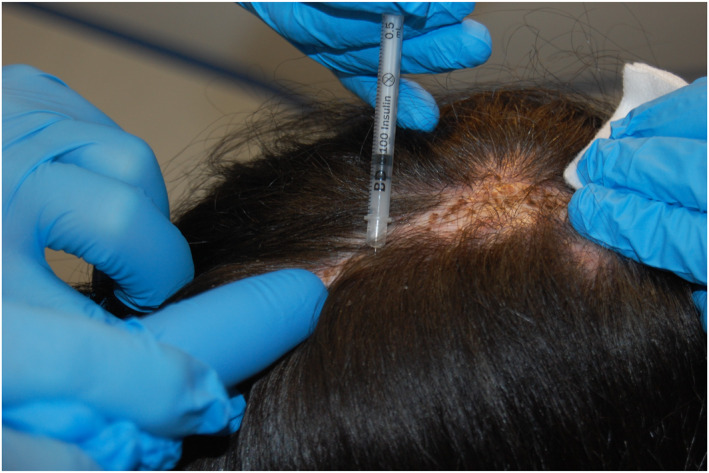

Dear Editor,

Intralesional corticosteroid therapy (ICT) is a recommended management strategy for various inflammatory hair loss disorders, including alopecia areata.^1^ Pain from ICT can limit the use of this treatment option in some patients, particularly younger people and those with needle phobia. However, strategies to mitigate pain during injections are rarely used in routine dermatology practice.

To investigate the efficacy and safety of vibration assisted analgesia (VAA), we audited pain‐perception immediately following ICT for alopecia, from our tertiary hair clinic, between March and August 2022, using a standard proforma and questionnaire. Vibration was delivered using a commercially available vibrator, placed inside a glove and applied to the nearby skin, as shown in Figure 1. ICT was delivered using 0.5–1 mL insulin syringes, with permanently attached 30 gauge needles. Small, re‐chargeable ‘bullet style’ vibrating devices were purchased from online retailers such as amazon or adult themed websites. No specific oscillation parameters were selected nor were they measured during the study. Other studies using VAA have quoted device frequencies between 150 and 183 Hz.^2^, ^3^

**FIGURE 1:**
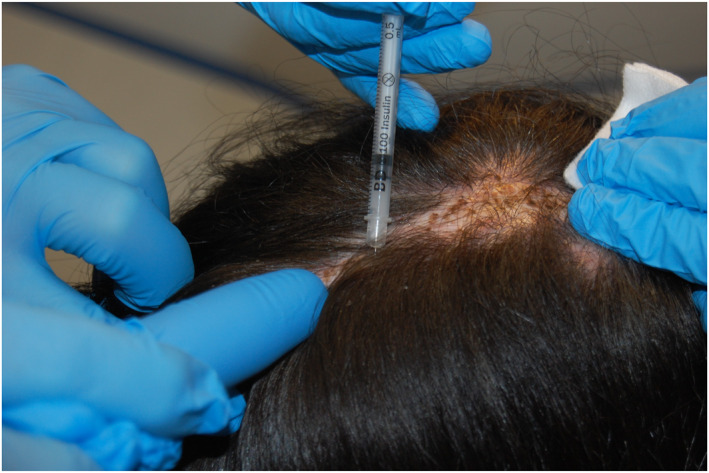
A gloved vibrator is placed roughly 2 cm from the site of intralesional corticosteroid therapy.

Fifty‐two patients were included (45 female: 7 male), with an average age of 47 years (range 16–85). The conditions treated included alopecia areata (*n* = 25), frontal fibrosing alopecia (*n* = 21), lichen planopilaris (*n* = 4), and folliculitis decalvans (*n* = 2).

Forty‐nine/52 patients received VAA during ICT. Of these, 39/49 had previously received, and could recall, their experiences with ICT without analgesia, allowing for comparison of pain. Of these: 85% (*n* = 33) felt that their pain was improved, with *n* = 23 stating this was ‘a lot better’ and *n* = 10 ‘a little better’; 13% (*n* = 5) felt VAA ‘made no difference’; and one person felt it was ‘a little worse’. One patient felt VAA caused a headache. No other adverse events were reported.

While topical local anaesthetics such as EMLA^®^ can reduce pin prick sensation, they are less effective at reducing pain induced by injection of material into the intradermal/subcutaneous space (e.g., during local anaesthetic administration).^2^ The reduction of pain due to VAA may be explained by the gate control theory of pain, whereby in an ‘open position’, small diameter c fibres are able to transmit pain signals without inhibition. However, activation of larger diameter A‐beta fibres with vibration, results in activation of inhibitory interneurons in the substantia gelatinosa,^4^ reducing transmission of c fibre signals to ascending neurones in the spinal cord. Therefore, vibration results in a ‘closed position’ of the gate and reduces pain perception,^4^ not only from pinprick, but also from injections deeper into the skin.^5^

VAA has been used effectively during ICT for keloid scars^2^ and scalp mesotherapy for alopecia,^3^ but comparative data for ICT in alopecia is lacking. Our data and ongoing experience demonstrate that VAA is an easy to use, effective, inexpensive and safe technique. Unlike topical anaesthetic, it is effective in treating both the pain from injection as well as the pinprick sensation of the needle and does not require time to take effect. This has allowed us to use ICT in a wider cohort of patients, including children and those with widespread scalp alopecia, who would otherwise not tolerate this procedure.

## CONFLICT OF INTEREST STATEMENT

The authors declare no conflicts of interest.

## AUTHOR CONTRIBUTIONS

**Matthew D. Wynne**: Formal analysis (lead); writing—original draft (lead); writing—review and editing (equal). **Thomas Harries**: Data curation (lead). **Charlotte Hennegan**: Investigation (supporting); writing—review and editing (supporting). **Nuala O'Donoghue**: Investigation (supporting); writing—review and editing (supporting). **Donna M. Cummins**: Investigation (supporting); writing—review and editing (supporting). **Matthew Harries**: Conceptualization (lead); investigation (supporting); methodology (lead); supervision (lead); writing—review and editing (equal).

## FUNDING INFORMATION

NIHR Manchester Biomedical Research Centre, NIHR203308

## ETHICS STATEMENT

Not applicable.

## Data Availability

The data will be shared on reasonable request to the corresponding author, including the questionnaire used to assess pain responses.
